# A functionalized metal–organic framework decorated with O^–^ groups showing excellent performance for lead(ii) removal from aqueous solution†
†Electronic supplementary information (ESI) available. CCDC 1536031. For ESI and crystallographic data in CIF or other electronic format see DOI: 10.1039/c7sc03308g


**DOI:** 10.1039/c7sc03308g

**Published:** 2017-09-18

**Authors:** Caixia Yu, Zhichao Shao, Hongwei Hou

**Affiliations:** a College of Chemistry and Molecular Engineering , Zhengzhou University , Zhengzhou 450001 , P. R. China . Email: houhongw@zzu.edu.cn; b Henan Key Laboratory of New Optoelectronic Functional Materials , College of Chemistry and Chemical Engineering , Anyang Normal University , Anyang 455000 , P. R. China

## Abstract

Heavy metal ions are highly toxic and widely spread as environmental pollutants. New strategies are being developed to efficiently remove these toxic ions. Herein, we use the intrinsic advantages of metal–organic frameworks (MOFs) and develop a porous Zn(ii)-based MOF decorated with O^–^ groups for the removal of Pb^2+^. Benefiting from its multiple porosity, sufficient adsorption sites and strong affinity, the activated MOF material exhibits an ultrahigh Pb^2+^ uptake capacity (616.64 mg g^–1^), surpassing all those of reported MOF adsorbents. Moreover, it can selectively capture Pb^2+^ with high efficiency (>99.27%) against background ions. Even in the presence of a high concentration of competitive ions, such as Ca^2+^ or Mg^2+^, effective removal (>99.21%) can also be achieved in a short time. The excellent removal performance demonstrates the strong electrostatic attraction and coordination interaction between the highly accessible O^–^ groups and Pb^2+^. The possible adsorption mechanism was systematically verified by zeta potential, FT-IR and XPS studies. Our work reveals the enormous potential of functionalized MOFs as an appealing platform to construct sorbent materials.

## Abstract 2

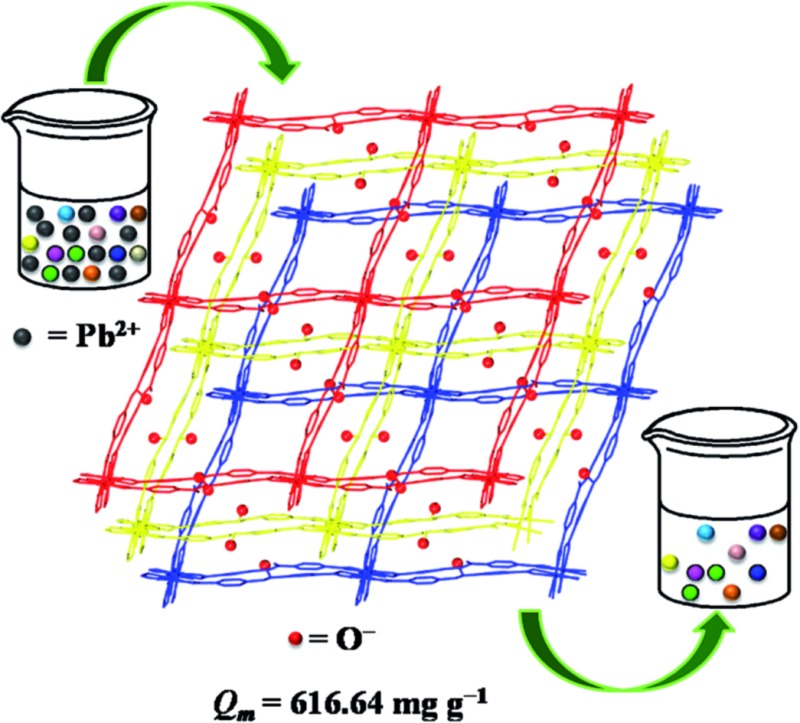
A novel MOF decorated with O^–^ groups was elaborately constructed and showed excellent performance for Pb^2+^ removal.

## Introduction

Lead (Pb^2+^), a prevalent and bio-accumulative heavy metal, has been recognized as one of the most toxic metals.^1^ With the rapid growth of industrialization and urbanization, Pb^2+^ discharge has become a serious environmental concern.^2^ At present, available clean water is limited and 90% of the available fresh water will be consumed by 2025.^3^ Almost all countries strive to remove Pb^2+^ from potable water in order to reduce levels of elevated blood Pb^2+^ in children.^4^ Owing to this fact, the removal of Pb^2+^ from water is critical in terms of the protection of public health and the environment. Many efforts have been dedicated to purify wastewater to get more available clean water. However, an efficient, cost-effective, robust and handy technology for the decontamination of water is urgently needed. The adsorption technique for removing heavy metal ions has gained extensive attention considering the easy operation, eco-friendliness and cost-effectiveness.^1^,^5^ Some effective adsorbents have been continuously developed and improved, including zeolites,^6^ carbon materials,^7^ clay minerals,^8^ nanomaterials^9^,^10^ and chelating polymers.^11^ Meanwhile, those sorbents face some challenges, such as a low capacity, a moderate affinity/selectivity, difficulties in separation, and a lack of structural and functional tunability,^12^ which have largely limited the effectiveness for Pb^2+^ removal. Therefore, there is an increasing interest in developing more efficient absorbents for the removal of Pb^2+^ from aqueous solution.

Metal–organic frameworks (MOFs), constructed by metal ions or metal clusters and organic ligands through coordination bonds, are considered as a favorable platform for adsorption applications,^13^–^15^ because of their high surface area, tunable chemical composition, variable pore size distribution and exposed active sites.^16^–^18^ By ligand modifications or MOF post-functionalization, various functional groups can be purposefully incorporated into the pores of MOFs, giving rise to more active sites for facile adsorption. The functionalized MOFs decorated with neutral groups, such as thiol/thioether/hydroxyl/azine/sulphur-functional groups, were exploited for the removal of Pb^2+^, UO_2_^+^, Cd^2+^, Hg^2+^, *etc.* from aqueous solution.^5^,^19^–^29^ Two elaborately constructed sulphur-functionalized MOFs, FJI-H9 and FJI-H12, could selectively remove Cd^2+^ and Hg^2+^ from water with high uptake capacities (286 mg g^–1^ for Cd^2+^ and 439.8 mg g^–1^ for Hg^2+^).^28^,^29^ Nonetheless, the introduction of negatively charged groups into MOFs for the highly efficient removal of heavy metal ions has rarely been reported.

**Scheme 1:**
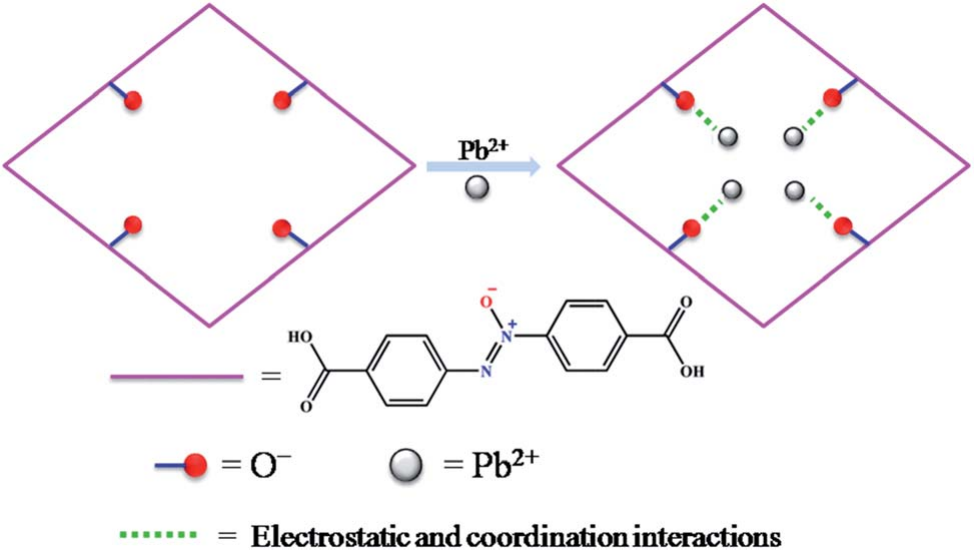
Schematic illustration of functionalized MOF for Pb^2+^ capture.

Herein, a functionalized MOF decorated with negatively charged O^–^ groups was designed and applied to the removal of Pb^2+^ from aqueous solution (Scheme 1). Based on the design, the negative charges of the O^–^ groups generate electrostatic interaction with Pb^2+^, which acts as a driving force in the adsorption process. Secondly, the O^–^ groups also worked as active sites to form coordination bonds with Pb^2+^. Thirdly, as a Lewis acid, Pb^2+^ has a lower hydration energy and a larger ionic radius, and could readily accept electrons from the Lewis base of the O^–^ groups. As a result, the O^–^ groups show a significant affinity and high selectivity for Pb^2+^. Fourthly, the multiple porosity densely populated with O^–^ groups would endow Pb^2+^ with fast sorption and a high removal efficiency. In this study, we synthesized a three-dimensional (3D) porous framework {[Zn_3_L_3_(BPE)_1.5_]·4.5DMF}_*n*_ (**1**, H_2_L = 4,4′-azoxydibenzoic acid, BPE = bis(4-pyridyl)ethylene, DMF = *N*,*N*-dimethylformamide) functionalized with O^–^ groups for the removal of Pb^2+^. The activated MOF material [Zn_3_L_3_(BPE)_1.5_]_*n*_ (**1a**) exhibited an ultrahigh uptake capacity (616.64 mg g^–1^), a very high affinity (*K*_d_ 10^6^ mL g^–1^) and a high removal efficiency for Pb^2+^. The adsorption mechanism was revealed by zeta potential, FT-IR and XPS studies. To the best of our knowledge, we are the first to introduce functional groups with negative charges into the pores of MOFs for the removal of heavy metal ions with excellent performance.

## Experimental

### Materials and apparatus

The ligand H_2_L was synthesized according to the literature method.^30^ All of the other reagents and chemicals were of an analytical grade and obtained from commercial sources. Powder X-ray diffraction (PXRD) data were collected on a PANalytical X’Pert PRO MPD system (PW3040/60). Fourier transform infrared (FT-IR) measurements were conducted on a Thermo Nicolet iS50 spectrometer. Scanning electron microscopy (SEM) images were taken on a Hitachi SU8010 instrument. X-ray photoelectron spectroscopy (XPS) data were obtained with a Thermo Escalab 250 spectrometer with monochromated Al-Kα excitation. The zeta potentials were determined using dynamic light scattering (DLS) on a Malvern Instruments Nanosizer-ZS. Thermogravimetric analysis (TGA) was carried out on a Netzsch STA-449F3 thermogravimetric analyzer under a nitrogen atmosphere at a heating rate of 10 °C min^–1^. Simultaneous inductively coupled plasma optical emission spectrometry (ICP-OES) on a PerkinElmer Optima 8000 instrument was used to determine the metal ion concentration in aqueous solution.

### Preparation of {[Zn_3_L_3_(BPE)_1.5_]·4.5DMF}_*n*_ (**1**)

Zn(NO_3_)_2_·6H_2_O (14.9 mg, 0.05 mmol), H_2_L (14.3 mg, 0.05 mmol), BPE (4.6 mg, 0.025 mmol) and 6 mL DMF were placed in a 10 mL vial. The mixture was stirred until complete dissolution, and then it was kept in an oven at a temperature of 100 °C for 24 h. Orange block crystals of **1** were obtained, washed with DMF, and dried at room temperature. Yield: 8.8 mg (40%, based on H_2_L). Anal. calcd for C_73.5_H_70.5_N_13.5_Zn_3_O_19.5_: C, 53.46; H, 4.30; N, 11.45. Found: C, 53.38; H, 4.07; N, 11.07.

### Preparation of [Zn_3_L_3_(BPE)_1.5_]_*n*_ (**1a**, activated **1**)

MOF **1a** was prepared by heating MOF **1** at 140 °C under vacuum (24 h) to remove the encapsulated DMF guests before the adsorption studies. As confirmed by PXRD, TG and elemental analysis, **1a** retained the same framework of **1**, but without DMF solvent molecules in the cavity. Anal. calcd for C_60_H_39_N_9_Zn_3_O_15_: C, 54.50; H, 2.97; N, 9.53. Found: C, 54.21; H, 3.22; N, 9.15.

### X-ray data collection and structure determination

Single X-ray diffraction intensities of crystals were collected on a CCD diffractometer at 153 K. All diffractometers were equipped with graphite monochromated Mo-Kα radiation (*λ* = 0.71073). The structure was solved by a direct method and expanded with the Fourier technique. All of the calculations were performed with the SHELXL-97 package.^31^ In **1**, two azoxy groups of the L^2–^ ligand were found to be disordered over two positions. All H atoms in **1** were placed in geometrically idealized positions and constrained to ride on their parent atoms. Moreover, the diffused electron densities resulting from these residual solvent molecules were removed from the data set using the SQUEEZE routine of PLATON and refined further using the data generated.^32^ The formula of {[Zn_3_L_3_(BPE)_1.5_]·4.5DMF}_*n*_ was derived from thermogravimetric characterization. The crystal data for **1** are summarized as follows: C_60_H_39_N_9_O_15_Zn_3_, *M*_r_ = 1322.17, monoclinic, space group *C*2/*c*, *a* = 19.604(4) Å, *b* = 28.079(6) Å, *c* = 32.670(7) Å, *α* = 90°, *β* = 94.47(3)°, *γ* = 90°, *V* = 17 929(7) Å^3^, *Z* = 8, *D*_c_ = 0.980 g cm^–3^, *F*(000) = 5376 and *μ* = 0.846 mm^–1^, 84 732 reflections collected, 15 810 unique (*R*_int_ = 0.0546). *R*_1_ = 0.0623, w*R*_2_ = 0.1871 and *S* = 1.093. Crystallographic data have been submitted to the Cambridge Structural Database with the deposition number CCDC ; 1536031.†

### Adsorption studies

All of the adsorption experiments were carried out at 25 °C using 10 mg of MOF **1a** and 80 mL of Pb^2+^ standard solution. The Pb^2+^ solution was prepared by dissolving Pb(NO_3_)_2_ in deionized water and diluting to the desired concentration. To study the adsorption kinetics, adsorption experiments were performed at pH 6.0 (10 ppm Pb^2+^ solution) under continuous stirring. The Pb^2+^ concentrations of samples were measured at given time intervals using an ICP-OES spectrometer. The adsorption isotherm experiments were investigated by adding 10 mg MOF **1a** into 80 mL Pb^2+^ solutions with different concentrations to reach adsorption equilibrium within 180 min. The adsorption capacity *q*_*t*_ (mg g^–1^) and the removal efficiency were obtained from the following equations:
1

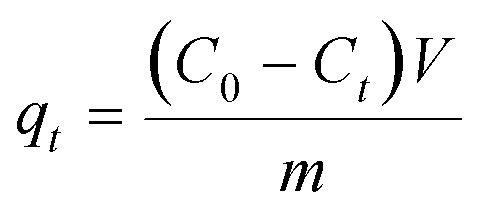



2

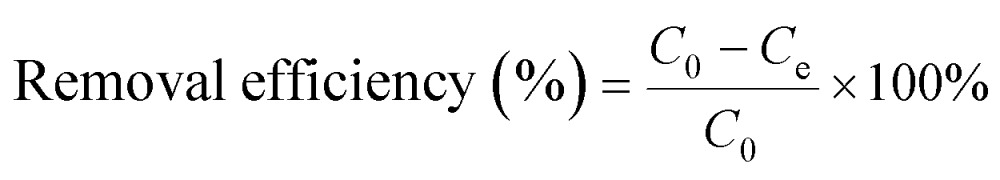

where *C*_0_ (mg L^–1^) and *C*_e_ (mg L^–1^) are the initial and equilibrium Pb^2+^ concentrations, respectively; *C*_*t*_ (mg L^–1^) is the Pb^2+^ concentration at time *t* (min); *V* (L) is the volume of the solution, and *m* (g) is the dry weight of adsorbent.

Furthermore, the desorption test was carried out by immersing the Pb^2+^ loaded MOF in 80 mL of HNO_3_ solution (0.1 mmol L^–1^) at 25 °C for 24 h. Then it was washed three times with water and dried under vacuum at 140 °C for 24 h. The desorbed Pb^2+^ was evaluated by the same method used in the adsorption studies.

## Results and discussion

### Characterization of **1** and **1a**

**Fig. 1:**
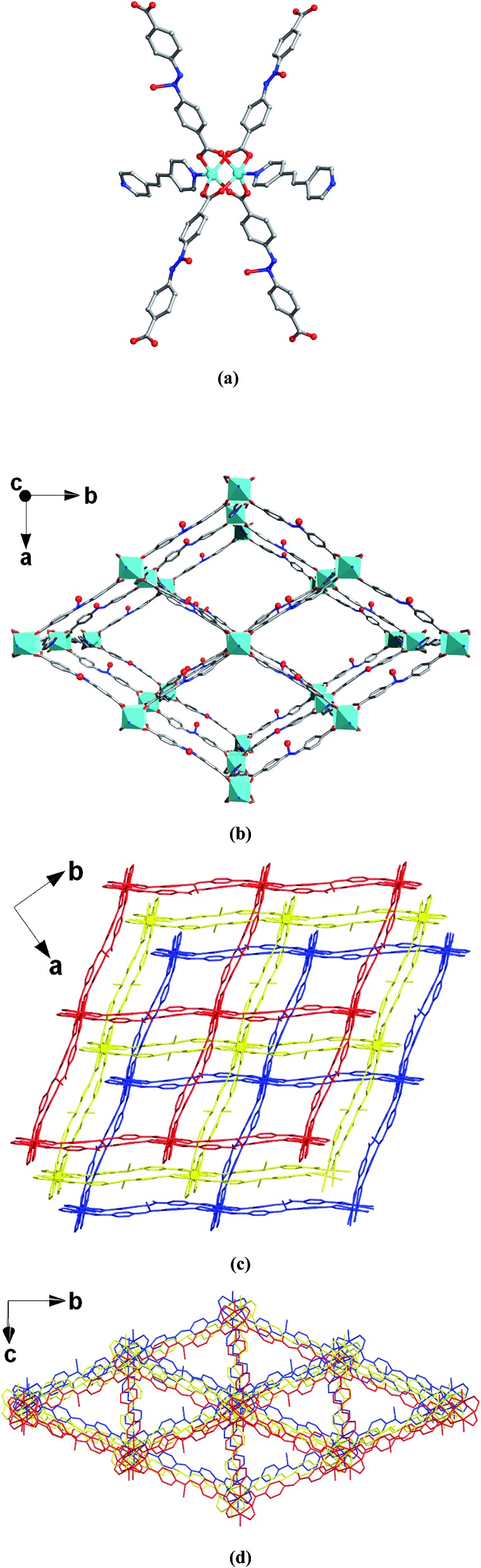
(a) View of the paddle-wheel building unit and the coordination environment of the Zn centers in **1**. (b) View of the 3D framework with 1D channels in **1** looking down the *c* axis. Atom color codes: Zn, cyan; O, red; N, blue; C, gray. (c and d) Two 1D channels of **1** viewed along two different directions.

The single-crystal X-ray diffraction study indicates that MOF **1** crystallizes in the *C*2/*c* monoclinic space group. The framework is composed of paddlewheel dinuclear Zn_2_(COO)_4_ secondary building units that are bridged by L^2–^ ligands and further pillared by BPE to construct an interpenetrating 3D framework (Fig. 1). Although interpenetration occurs, two kinds of pore are still present in MOF **1** (Fig. 1c and d), which are calculated by the Platon program^31^ to have an effective solvent accessible volume of 8462.8 Å^3^ per unit cell (47.2% of the total cell volume). As expected, the pores in MOF **1** are decorated by a large number of O^–^ sites, resulting in the functionalized pores. These functionalized pores will certainly be favorable for the capture of heavy metal ions.

Thermogravimetric analysis (TGA) of **1** displays a major weight loss (20.2%) of the DMF guest molecules at 40–222 °C (Fig. S1†). So, the as-synthesized **1** was heated at 140 °C under vacuum for 24 h to generate the activated sample **1a**. The TGA of **1a** showed that the DMF molecules were completely removed (Fig. S1†), which suggested it would be a potential sorbent. The TGA, together with the elemental analysis, confirmed that the chemical formula of **1a** was [Zn_3_L_3_(BPE)_1.5_]_*n*_.

To examine the permanent porosity of **1a**, gas sorption isotherms were investigated (Fig. S2†). Unexpectedly, there is almost no N_2_ adsorption at 77 K compared to CO_2_ adsorption at 195 K, which can possibly be attributed to the different kinetic diameters of N_2_ (3.64 Å) and CO_2_ (3.30 Å which results in easier diffusion into the micropores). Similar N_2_ and CO_2_ sorption behaviors have been observed in many other flexible MOF materials.^33^–^38^ The Brunauer–Emmett–Teller surface area of **1a** is calculated to be 82.5 m^2^ g^–1^ based on the CO_2_ adsorption isotherm. This is probably due to a higher diffusion barrier imposed on the 1D pore for the flexible MOF after desolvation and the corresponding structural contraction.^33^,^34^

As illustrated in Fig. S3,† the X-ray diffraction peaks obtained from **1a** were sharp, indicating the crystalline nature of the desolvated phase. The low-angle Bragg’s reflection in **1** vanished in **1a**. According to the literature, there is a slight shrinkage of the framework of **1a** after guest molecule removal from **1**.^38^–^41^ Soaking **1a** in DMF for 24 h generated **1′**, the low-angle Bragg’s reflection reappeared and the PXRD pattern was almost the same as that of **1** (Fig. S3†), which indicates that **1a** is a flexible MOF and the framework did not collapse during this process. These results can be explained by the breathing behavior which has been extensively studied over the past few decades (Scheme S1†).^34^,^35^,^41^,^42^ For the flexible interpenetrated frameworks, subtle differences of guest content and composition will lead to different structures, and this transformation is reversible.^33^,^36^,^39^,^43^,^44^ Interestingly, a slight difference was observed in the PXRD data simulated from the single crystal data of **1** at 153 K and collected at 298 K (Fig. S3†). It can be concluded that the temperature induced structural changes in the flexible interpenetrated frameworks^34^,^39^ and also resulted in the different unit cell parameters (153 K, *a* = 19.604 Å, *b* = 28.079 Å, *c* = 32.670 Å, *α* = 90°, *β* = 94.47°, *γ* = 90°, *V* = 17 929 Å^3^; 298 K, *a* = 21.79 Å, *b* = 26.94 Å, *c* = 33.00 Å, *α* = 90°, *β* = 92.24°, *γ* = 90°, *V* = 19 358 Å^3^).

To elucidate the solvent induced breathing behavior,^33^**1** was immersed in water (24 h), and the single crystallinity of the resulted H_2_O-exchanged **1** (**1**–H_2_O) was not good. However, by immersing **1**–H_2_O in DMF for 24 h, **1**–H_2_O–DMF with better crystal quality was obtained and the unit cell parameters were very similar to those of **1** (298 K, Table S1†). So the structural transformation is reversible, and it is directly evidenced by the single crystal images (Fig. S4†). To reveal the structural change in the process of solvent-exchange, we tried to determine the crystal structures of various solvent-exchanged MOFs. Fortunately, we got the single-crystal structure of CHCl_3_-exchanged **1** (**1**–CHCl_3_) at 153 K (Table S2†), which retained the original metal–ligand connectivity. Meanwhile the total cell volume decreased from 17 929 Å^3^ for **1** (153 K) to 17 753 Å^3^ for **1**–CHCl_3_ (Table S2†), indicating a slight contraction of the square grid (Fig. S5†). After soaking **1**–CHCl_3_ in DMF, the unit cell parameters of the generated **1**–CHCl_3_–DMF were almost the same as those of **1** (298 K). These results further confirmed that the MOF is dynamic in nature and the transformation of the flexible framework is reversible,^33^,^37^,^39^,^43^ which is also supported by the PXRD patterns. As shown in Fig. S6,† the PXRD patterns of solvent-exchanged **1** are different from those of **1**, but return to **1** after immersion in DMF for 24 h (Fig. S7†).

### Pb^2+^ sorption studies

The pH value greatly influences the adsorption performance of Pb^2+^, so the effect of the pH ranging from 3.0 to 7.0 on the adsorption of Pb^2+^ was investigated. As shown in Fig. S8,† the removal efficiency of **1a** was very low at pH 3.0 and it increased dramatically with the increasing of the pH and achieved the largest signal at pH 6.0. Further increasing the pH resulted in a decline of the removal efficiency. This is due to the protonation effect on the surface of the MOF adsorbent and excess H^+^ competing for the sorption sites at a low pH. When the pH is higher than 6.0, Pb^2+^ hydroxide precipitation may occur.^45^ Accordingly, the optimum pH value of 6.0 was selected for the subsequent adsorption experiments.

**Fig. 2:**
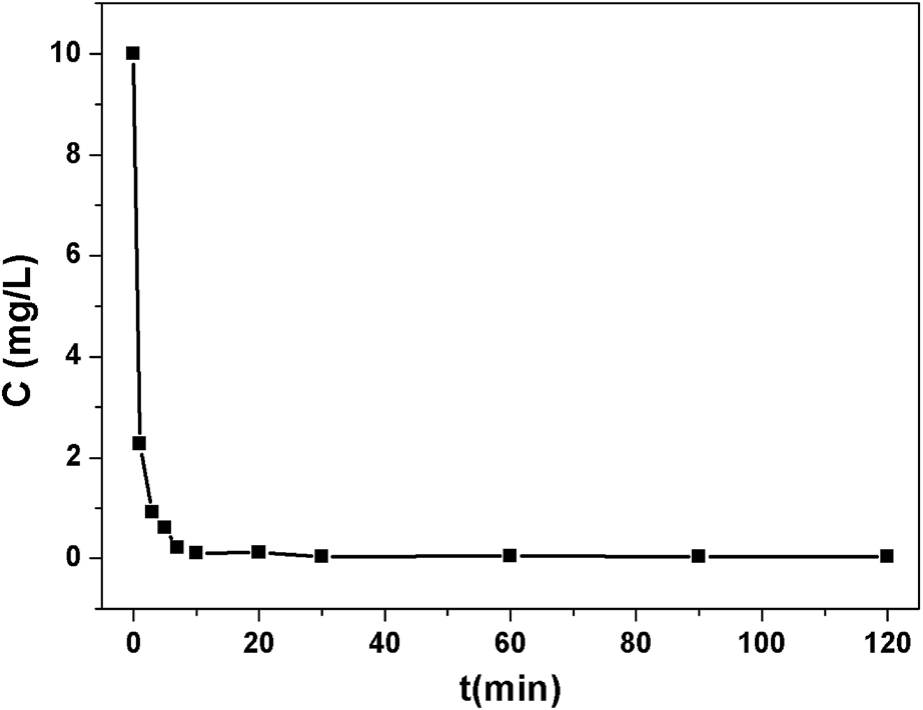
The kinetics and efficiency of **1a** for Pb^2+^ removal with an initial concentration of 10 ppm.

To evaluate the effectiveness of Pb^2+^ removal from water, **1a** was placed in a Pb^2+^ solution of 10 ppm at pH 6.0. The Pb^2+^ loaded **1a** (**1a**–Pb) was isolated and washed with water to remove the residual Pb^2+^ on the exterior of **1a**–Pb. Then it was examined by energy-dispersive X-ray spectroscopy (EDS) and this confirmed the existence of Pb (Fig. S9†). As shown in Fig. 2, **1a** can rapidly capture Pb^2+^, and remove 98.12% Pb^2+^ within 7 min. After 1 h, the concentration of the residual Pb^2+^ reduced to 0.035 ppm, that is, 99.65% Pb^2+^ was removed. The fast kinetics and high efficiency for Pb^2+^ removal could be attributed to the high affinity of **1a**.

To assess the sorbent’s affinity for Pb^2+^, the distribution coefficient *K*_d_ (mL g^–1^) was calculated as follows:
3

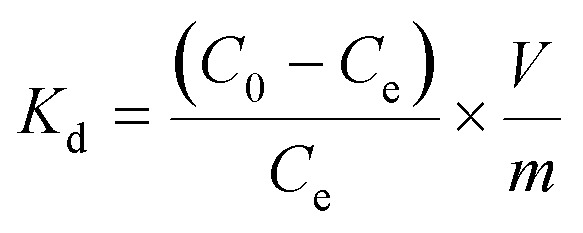

where *C*_0_ (mg L^–1^) and *C*_e_ (mg L^–1^) are the initial and equilibrium concentrations of Pb^2+^; *V* is the volume of the treated solution (mL); *m* is the dry weight of adsorbent (g). *K*_d_ represents an important aspect of the sorbent performance. In general, a material with a *K*_d_ value above 10^4^ mL g^–1^ is considered to be an excellent adsorbent.^46^,^47^ The *K*_d_ value of **1a** for Pb^2+^ is 2.3 × 10^6^ mL g^–1^, surpassing that of various reported benchmark materials, for examples, biochars (10^3^ to 10^4^ mL g^–1^),^48^ mesoporous carbon (6.82 × 10^5^ mL g^–1^),^49^ commercial resins (10^4^ to 5.1 × 10^5^ mL g^–1^),^50^ lignin functionalized carbon nanotubes (3.6 × 10^5^ mL g^–1^),^51^ MoS_4_-LDH (2.6 × 10^5^ mL g^–1^),^47^ and pampeano aquifer (7.5 × 10^3^ to 1 × 10^4^ mL g^–1^).^52^

**Fig. 3:**
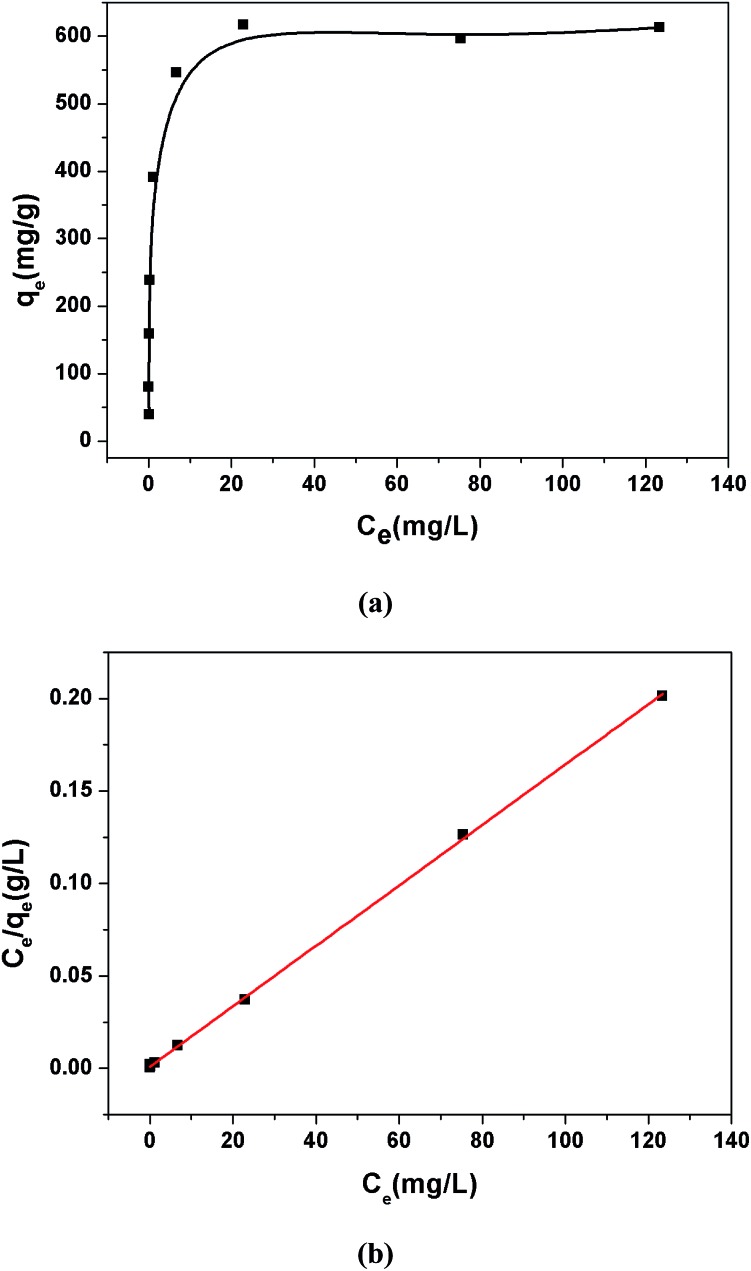
(a) Pb^2+^ adsorption isotherm for **1a**. (b) Langmuir adsorption mode fitting for the adsorption of Pb^2+^ by **1a**.

The adsorption isotherms were investigated to estimate the maximum adsorption capacity of **1a** by varying the Pb^2+^ concentrations from 5 ppm to 200 ppm at pH 6.0. As shown in Fig. 3a, the value of *q*_e_ (equilibrium adsorption capacity) increased with the increasing Pb^2+^ concentrations and finally reached the maximum value of 616.64 mg g^–1^. In addition, we also considered the removal efficiency at different Pb^2+^ initial concentrations (Fig. S10†). More than 90% Pb^2+^ can be removed in the concentration range from 1 ppm to 75 ppm. The equilibrium adsorption isotherm data was fitted by the Langmuir model, yielding a relatively high correlation coefficient of 0.9997. The maximum adsorption capacity of **1a** for Pb^2+^ was calculated to be 613.50 mg g^–1^, which closely matched with the experimental equilibrium value of 616.64 mg g^–1^. We reasoned that such an excellent lead adsorption capacity could be stemmed from the highly accessible O^–^ groups densely populated throughout the pores of **1a**.

To highlight the contribution of the O^–^ groups to the Pb^2+^ uptake, we synthesized a similar MOF to **1** but without O^–^ groups, [Zn(ADC)(BPE)_0.5_]_*n*_ (ADC = 4,4′-azobenzenedicarboxylate),^37^ and investigated its adsorption isotherms for Pb^2+^. Results show that the activated [Zn(ADC)(BPE)_0.5_]_*n*_ displayed a lower uptake capacity of 473.92 mg g^–1^. Meanwhile, we calculated the theoretical uptake capacity of **1a** based on the molecular formula, pore volumes and one O^–^ group per L^2–^ adsorbing one Pb^2+^ ion by a coordination interaction. The calculated theoretical uptake capacity is 470.11 mg g^–1^, which is obviously lower than the experimental equilibrium value (616.64 mg g^–1^) of **1a**. The higher experimental adsorption capacity for **1a** could be attributed to the various adsorption modes formed by the O^–^ groups, which offered not only coordination interactions but also electrostatic attractions between the O^–^ groups and Pb^2+^. To the best of our knowledge, the Pb^2+^ uptake capacity of **1a** is the highest among the reported MOF adsorbents (Table S3†), and also exceeded that of other Pb^2+^ adsorbents.^51^,^53^–^57^ These results suggest that **1a** is a prospective adsorbent for Pb^2+^ removal due to its large adsorption capacity and high removal efficiency.

**Fig. 4:**
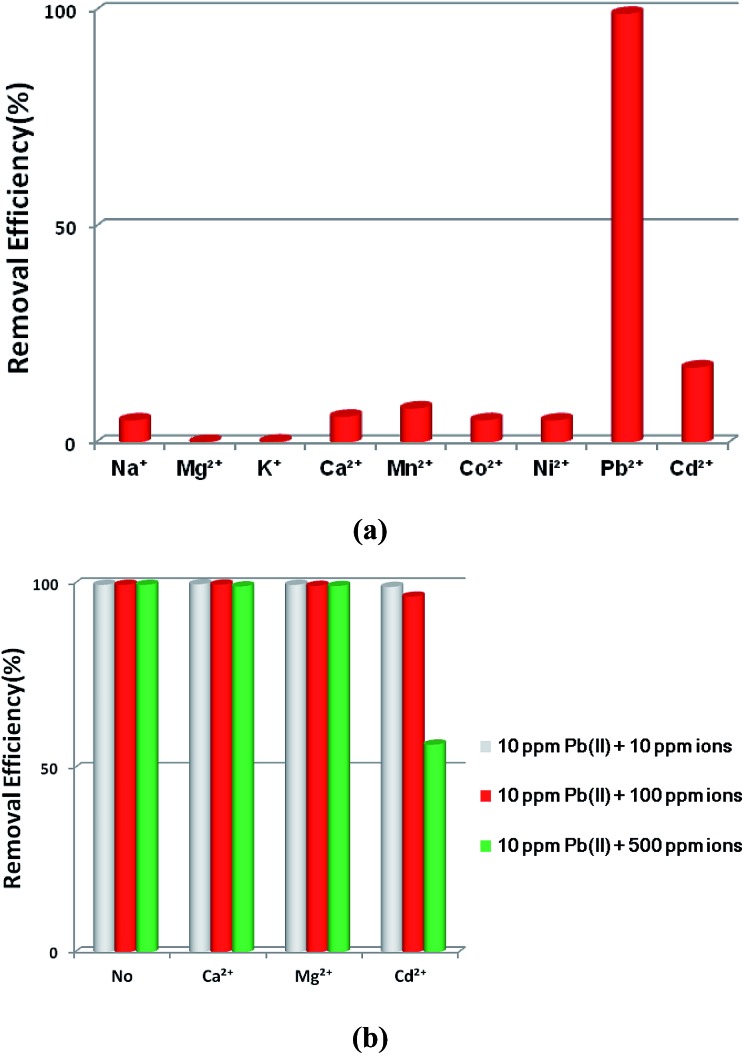
(a) The effects of coexisting ions on the removal of Pb^2+^ by **1a**. (b) The effects of competing ions on the removal of Pb^2+^ by **1a**.

The selective removal of heavy metal ions will facilitate environmental protection and allow the reuse of heavy metals.^53^ However, a lot of adsorbents for Pb^2+^ removal exhibit low selectivity. An absorbent with high selectivity is required to separate heavy metal ions from wastewater. Selectivity tests were performed in a mixed solution containing Na^+^, Mg^2+^, K^+^, Ca^2+^, Mn^2+^, Co^2+^, Ni^2+^, Cd^2+^ and Pb^2+^ with the concentration of 10 ppm for each metal ion. The adsorption ability of **1a** toward Pb^2+^ is considerably higher than that for other ions (Fig. 4a). In the mixed solution, 99.27% Pb^2+^ was removed, while 17.46% Cd^2+^ was absorbed. In contrast, other background metal ions such as Na^+^, Mg^2+^, K^+^, Ca^2+^, Mn^2+^, Co^2+^, and Ni^2+^ do not quite bind to **1a**, with an equilibrium removal efficiency of less than 8%. This phenomenon can be explained by the HSAB principle.^58^ The negatively charged O atoms of the azoxy groups have the properties of a borderline base, which could readily bind with the metals Co^2+^, Ni^2+^, and Pb^2+^ (borderline acids) and Cd^2+^ (soft acid), and bind difficultly with Na^+^, Mg^2+^, K^+^, Ca^2+^, and Mn^2+^ (hard acids). Compared to the borderline acid Pb^2+^, the relatively low removal efficiencies for the borderline acids Co^2+^ and Ni^2+^ are attributed to their fairly high hydration energies (1915 kJ mol^–1^ for Co^2+^, 1980 kJ mol^–1^ for Ni^2+^, and 1425 kJ mol^–1^ for Pb^2+^).^59^ It is difficult for Co^2+^ and Ni^2+^ to detach water molecules and further interact with the negatively charged O atoms. So the MOF material has a strong preference and fairly high selectivity for Pb^2+^ against other metal ions, highlighting its potential in selectively removing Pb^2+^ from real wastewater.

Ca^2+^ and Mg^2+^ are common cations that always ubiquitously co-exist in the natural water environment, and the same positive-charge and high level concentration might lead to competitive adsorption with Pb^2+^.^2^,^60^,^61^ The soft acid Cd^2+^, which has a comparable ionic size and charge density to Pb^2+^, and often co-exists with Pb^2+^,^62^ can strongly compete for the selectivity of sorbents. Thus, it is essential to investigate the effect of the competing ions Ca^2+^, Mg^2+^ and Cd^2+^ on the adsorption of Pb^2+^. As shown in Fig. 4b, outstanding Pb^2+^ removal efficiencies were obtained in the presence of competing ions of Ca^2+^ and Mg^2+^. With the molar ratios of Ca^2+^/Pb^2+^ and Mg^2+^/Pb^2+^ ranging from 1 to 50, there were only little decreases for Pb^2+^ removal efficiencies, changing from 99.82% to 99.21% with Ca^2+^ and 99.65% to 99.31% with Mg^2+^. A large removal capacity (96.43%) was still obtained at a molar ratio of 10 for Cd^2+^/Pb^2+^. When the ratio was increased to 50, the Pb^2+^ removal efficiency reduced to 56.31%. The concentration of Cd^2+^ in the natural water environment can hardly reach such a high ratio, thus Cd^2+^ could not affect the Pb^2+^ removal performance in a practical application.

All of these results indicate that **1a** has a strong preference and an ultrahigh selectivity for Pb^2+^ sorption. The particular selectivity can be ascribed to the following reasons: (1) the densely populated O^–^ groups of the functionalized MOF provided strong interactions between O^–^ and Pb^2+^; (2) adsorbents preferentially adsorb divalent cations with a lower hydration energy in general, as metal ions have to detach a large share of hydrated water before they enter the smaller channels of the adsorbents.^59^,^60^,^63^,^64^ Compared with Ca^2+^ (1505 kJ mol^–1^), Mg^2+^ (1830 kJ mol^–1^) and Cd^2+^ (1755 kJ mol^–1^), the lowest Gibbs free energy of hydration was observed for Pb^2+^ (1425 kJ mol^–1^), indicating Pb^2+^ preferential adsorption than other divalent cations;^59^ (3) it has been reported that a larger ionic radius would be favorable for the interactions between metal ions and functional groups of the adsorbent.^65^–^67^ The radiuses of Ca^2+^, Mg^2+^ and Cd^2+^ are 0.100 nm, 0.072 nm and 0.095 nm, respectively, while the radius of Pb^2+^ is 0.119 nm. The larger radius would endow **1a** with quite good selectivity for Pb^2+^ adsorption.

### Stability and reusability study

**Fig. 5:**
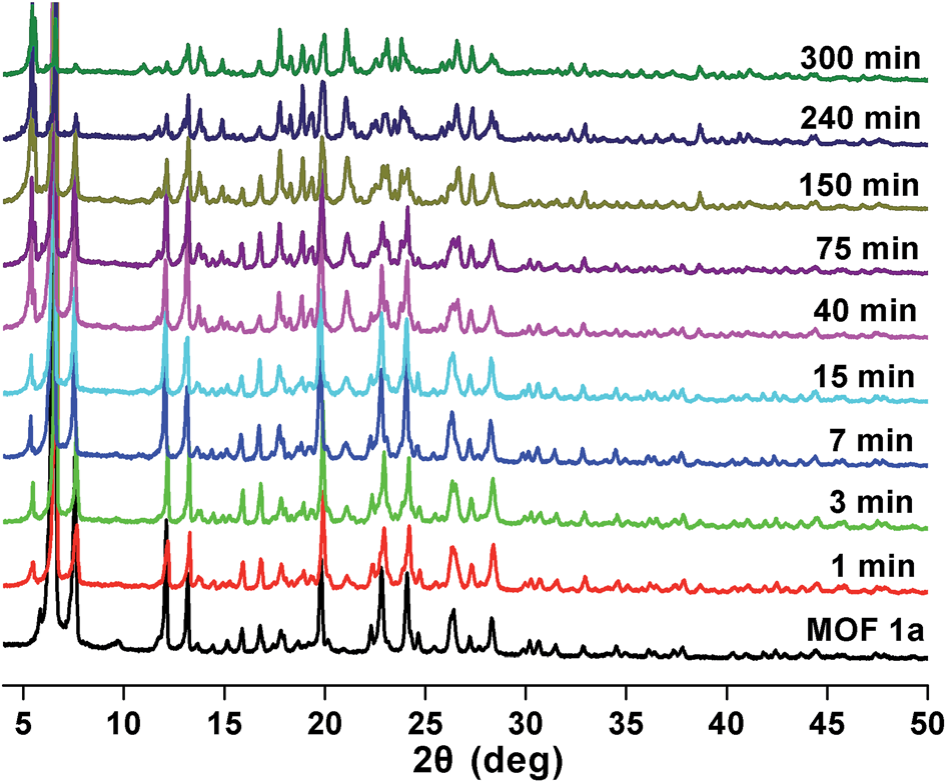
PXRD patterns of **1a** after soaking in water for different times.

Stability and reusability are two important factors in the application of an adsorbent. To verify the chemical stability of **1a**, we performed *in situ* PXRD measurements to evaluate the crystalline structure. In Fig. 5 and S11,† after soaking **1a** in water for 300 min, and even soaking in Pb^2+^ solution for more than 300 min, it still holds a high crystalline form and retained the structure of **1a**, indicating the good chemical stability of **1a** in water and Pb^2+^ solution. On further immersion of **1a** in aqueous solution of pH 3.0–10.0 (300 min), its high crystalline form was also preserved (Fig. S12†). Meanwhile, SEM investigations were carried out to examine the surface morphology of **1a** before and after loading Pb^2+^. As shown in Fig. S13b,† the crystals of **1a** did not collapse during the sorption process.^36^ The adsorption isotherm of **1a**–Pb exhibits a negligible uptake for CO_2_ (Fig. S14†). The different adsorption capacities and behaviors of **1a** and **1a**–Pb are clearly caused by loading Pb^2+^. To get single crystals with better quality, we soaked **1**–Pb in DMF for 24 h, and determined the unit cell parameters of **1**–Pb–DMF (Table S1†). By comparison of the unit cell parameters with **1** (298 K), we can affirm that the adsorbent still maintains the single crystallinity even after loading Pb^2+^.

**Fig. 6:**
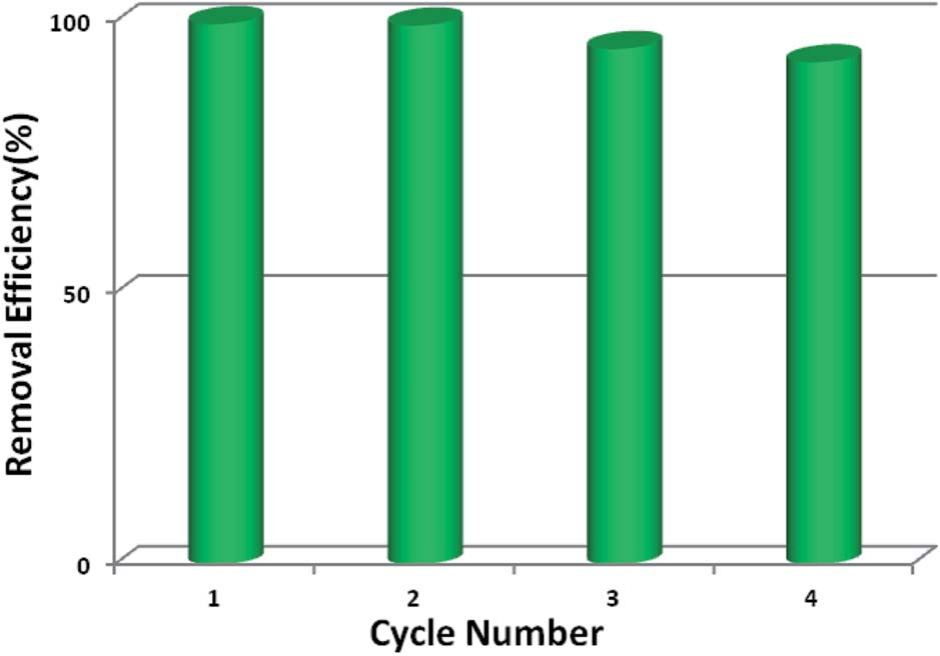
Reusability of **1a** for Pb^2+^ adsorption.

From the consideration of economical and practical purposes, it is of great significance to study the desorption and regeneration of an adsorbent. As shown in Fig. 6, **1a** exhibited a favorable cycle performance for Pb^2+^ adsorption, and the removal efficiency remained almost unchanged in the first two cycles and only slight fading was observed in the following cycles. The slight decrease may result from the loss of the adsorbent during cycling. Similar results were also reported in previous studies.^55^,^68^

### Mechanism of Pb^2+^ removal

Generally, the adsorptive modes for metal ion removal mainly include electrostatic adsorption, coordination adsorption and ion exchange. To investigate whether there is electrostatic attraction in the sorption process of Pb^2+^, zeta potential measurements were performed. When the pH was above 4.0, the surface of **1a** was negatively charged, and the density of the negative charge increased remarkably with increasing pH value (Fig. S15†). Meanwhile, the removal efficiency exhibited a similar variation tendency along with the change of pH value. The zeta potential of **1a**–Pb appeared to be less negative in the pH range of 5.0–7.0 due to charge neutralization. Hence, it is believed that the electrostatic attraction is the main force causing the adsorption of Pb^2+^ onto **1a**. When the solution pH was below 4.0, the surface charge on **1a** turned positive, resulting in electrostatic repulsion between **1a** and Pb^2+^. However, **1a** still exhibited a removal efficiency of 19.68% at a pH of 3.0, which demonstrates that there are other interactions besides the electrostatic effect.

Evaluating the effect of the background electrolyte on Pb^2+^ sorption is an effective macroscopic method to understand the adsorption mechanism.^69^ One can see that the background electrolyte had little effect on Pb^2+^ adsorption (Fig. S16†). Even at a high concentration of 500 ppm, a relatively high removal efficiency (98.39%) for Pb^2+^ was still obtained. Therefore, the mechanism of Pb^2+^ removal does not involve ion exchange behavior.

**Fig. 7:**
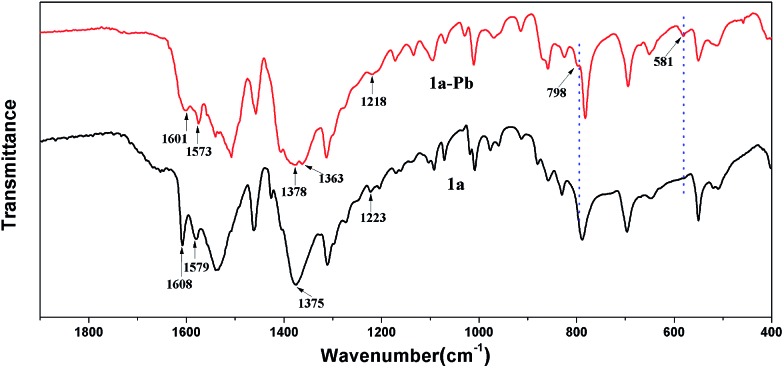
FT-IR spectra of **1a** and **1a**–Pb.

To further investigate the mechanism of Pb^2+^ sorption, FT-IR spectra of **1a** and **1a**–Pb were studied. In Fig. 7, a new peak at 581 cm^–1^ for **1a**–Pb is a typical characteristic stretching vibration of Pb–O,^70^ confirming the fact of Pb^2+^ loading. The bands at 1378 cm^–1^ and 798 cm^–1^ occur after Pb^2+^ adsorption, implying the presence of NO_3_^–^, for charge balance.^47^,^71^,^72^ There are strong interactions between Pb^2+^ and the O^–^ groups that could limit N–O stretching vibrations and consequently decrease their vibrational frequency, giving rise to a red shift from 1223 cm^–1^ to 1218 cm^–1^.^25^ For **1a**, the peaks at 1608 cm^–1^, 1579 cm^–1^ and 1375 cm^–1^ are assigned to the characteristic groups of the aromatic C=C and C–N vibrations,^73^ whereas these peaks shifted to 1601 cm^–1^, 1573 cm^–1^, and 1363 cm^–1^, respectively, after loading Pb^2+^. This might give further evidence of coordination interactions between Pb^2+^ and O atoms.

**Fig. 8:**
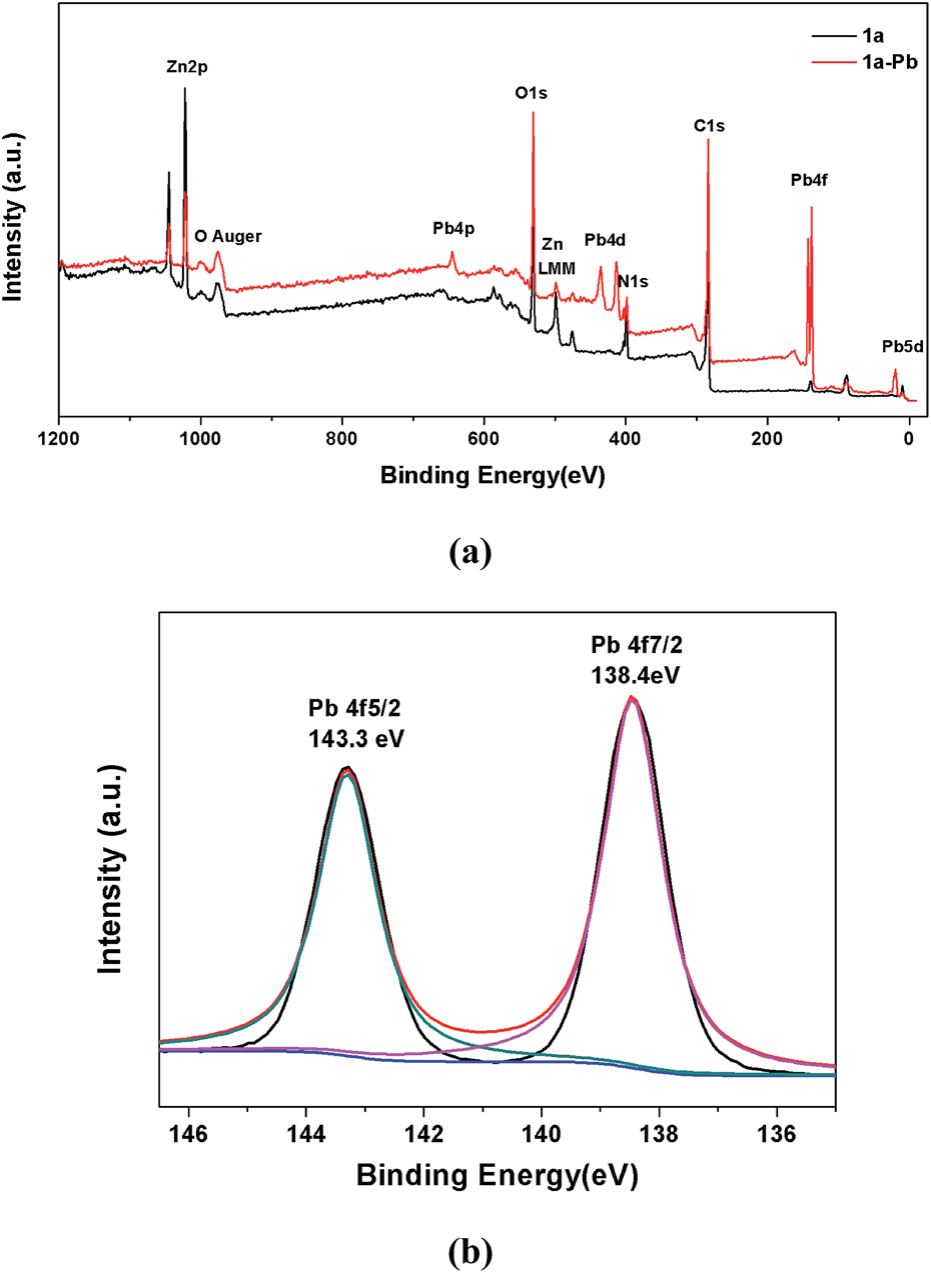
(a) XPS survey spectra of **1a** and **1a**–Pb. (b) Pb 4f XPS spectrum of **1a**–Pb.

XPS spectra were also employed to provide more information on the interactions between **1a** and Pb^2+^. As shown in Fig. 8a, the appearance of the Pb 4f, Pb 4d and Pb 4p peaks verifies that Pb^2+^ is undoubtedly loaded on **1a**. A more detailed structure of the Pb species could be obtained in the high resolution XPS spectrum of Pb 4f. In Fig. 8b, there are two peaks at 143.3 eV and 138.4 eV, corresponding to Pb 4f_5/2_ and Pb 4f_7/2_. Compared with the Pb^2+^ binding energies of purified Pb(NO_3_)_2_ at 144.5 eV for Pb 4f_5/2_ and 139.6 eV for Pb 4f_7/2_,^60^ a remarkable shift of 1.2 eV to a lower binding energy for Pb 4f can be observed in **1a**–Pb, which reveals the formation of strong affinities between Pb^2+^ and **1a**.^2^ The energy separation of 4.9 eV between the Pb 4f_5/2_ (143.3 eV) and Pb 4f_7/2_ (138.4 eV) peaks further confirms that the coordination interaction, and not merely the electrostatic interaction, accounts for the mechanism of Pb^2+^ sorption.^1^,^74^ These results are consistent with the FT-IR analyses and zeta potential measurements.

## Conclusions

In summary, a novel Zn(ii)-based MOF functionalized with O^–^ groups was elaborately constructed for the removal of Pb^2+^ from aqueous solution. The activated MOF showed excellent performance for Pb^2+^ removal with a record-high uptake capacity of 616.64 mg g^–1^ among MOF adsorbents, a high removal efficiency in a wide range (>90% at 1–75 ppm) and an exceptional distribution coefficient value of 2.3 × 10^6^ mL g^–1^, making the activated MOF one of the most promising materials for eliminating Pb^2+^ pollution from water. Notably, the activated MOF can selectively capture Pb^2+^ with a high efficiency even in the presence of a high concentration of other metal ions. Also, the adsorbent can be readily regenerated and recycled without a significant decrease of the removal efficiency. The densely populated and highly accessible O^–^ groups with their remarkable affinity for Pb^2+^ are responsible for the impressive results. This work therefore lays a foundation for introducing charged groups into MOFs as a new platform for efficiently removing contaminants from aqueous solution.

## Conflicts of interest

There are no conflicts to declare.

## Supplementary Material

Supplementary information

Crystal structure data
