# Supplementary material for: A functionalized metal–organic framework decorated with O– groups showing excellent performance for lead(ii) removal from aqueous solution
Source: Chem Sci. 2017 Sep 18;8(11):7611–9. doi: 10.1039/c7sc03308g (PMC5848797; doi:10.1039/c7sc03308g)
Supplement: Supplementary file 1 [file SC-008-C7SC03308G-s001.pdf]

Electronic Supplementary Material (ESI) for Chemical Science.
This journal is © The Royal Society of Chemistry 2017

Electronic Supplementary Information

A functionalized metal-organic framework decorated with O^- groups showing excellent performance for lead(II) removal from aqueous solution

Caixia Yu,[a,b] Zhichao Shao[a] and Hongwei Hou*[a]

[a] *College of Chemistry and Molecular Engineering, Zhengzhou University, Zhengzhou 450001, P. R. China*

[b] *Henan Key Laboratory of New Optoelectronic Functional Materials, College of Chemistry and Chemical Engineering, Anyang Normal University, Anyang 455000, P. R. China*

Synthesis of 4,4'-azoxydibenzoic acid (H_2L). *p*-Nitrobenzoic acid (3.20 g, 0.019 mol), 15.0 g 85% of potassium hydroxide (0.227 mol), and 150 mL of absolute MeOH were refluxed for 21 h. At the end of this time the MeOH was removed by evaporated under vacuum. This was stirred with 150 mL of water, filtered, and the remaining residue was discarded. The filtrate was adjusted to pH 7.0 with 10% HCl and the resultant precipitate was collected and air-dried to a crude products. The crude product was dissolved in dilute $NH_3 \cdot H_2O$ and reprecipitated with HCl. The precipitate was separated by filtered, washed with water, EtOH and air-dried, forming a yellow powder. Yield: 1.66 g (61%). The $\mathbf{H_2L}$ was characterized by ^{1}H NMR, ^{13}C NMR and MS (Figure S17).

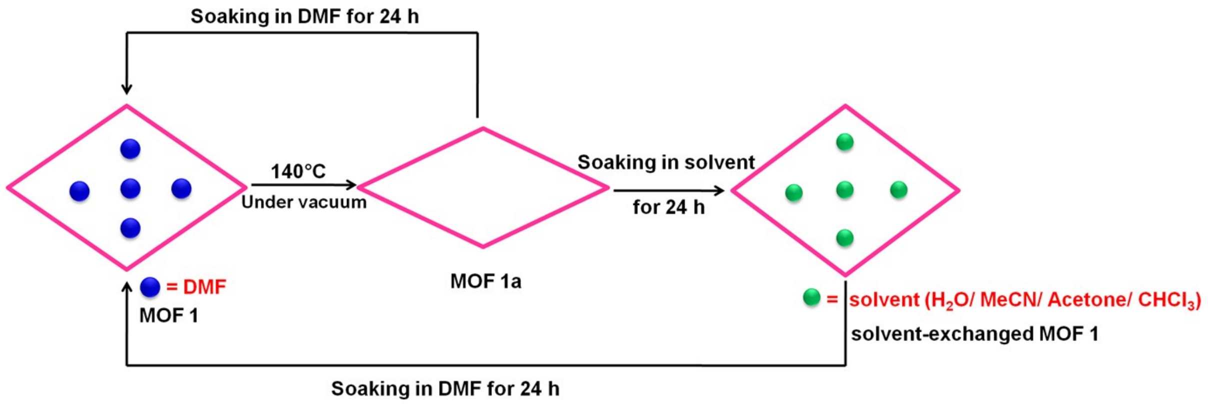

Scheme S1. Schematic illustration of the breathing behavior.

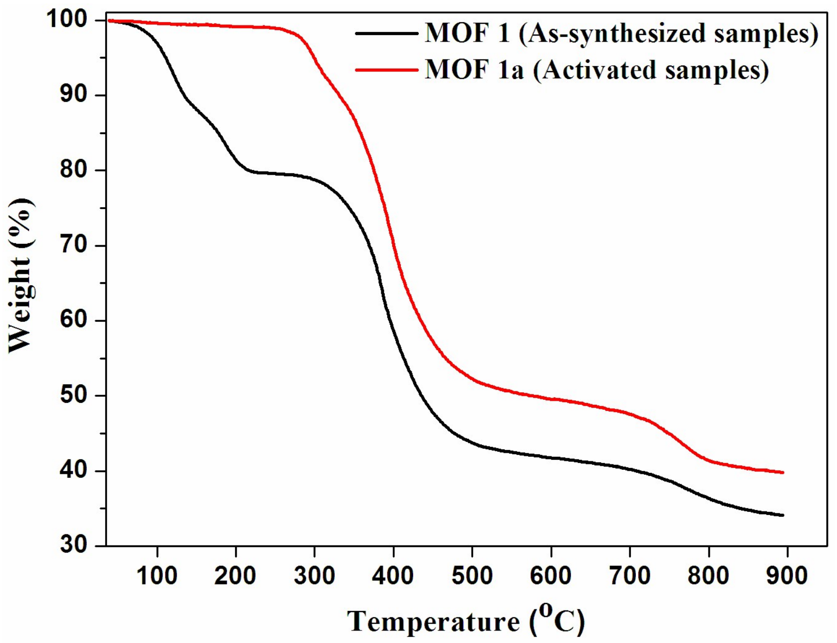

Figure S1. TGA curves of **1** and **1a**.

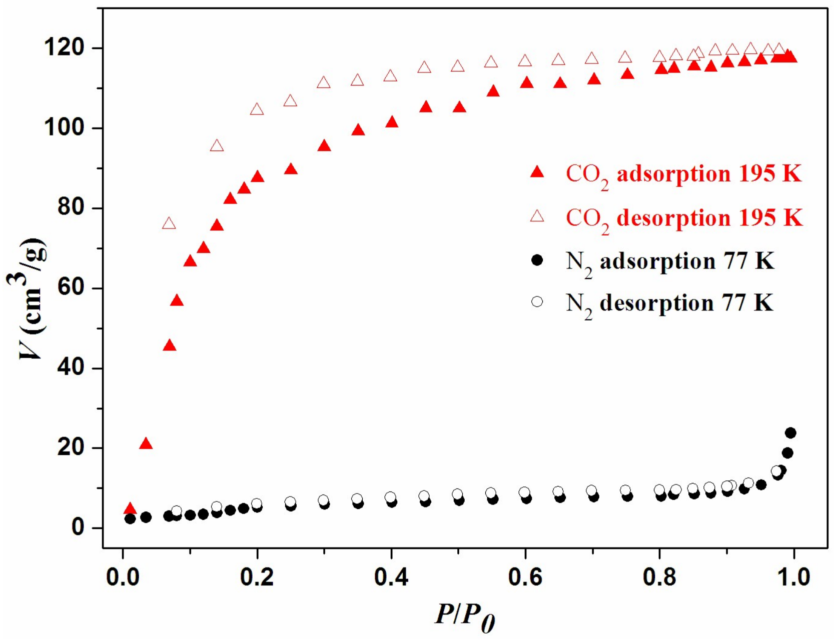

Figure S2. Gas adsorption and desorption isotherms of **1a**.

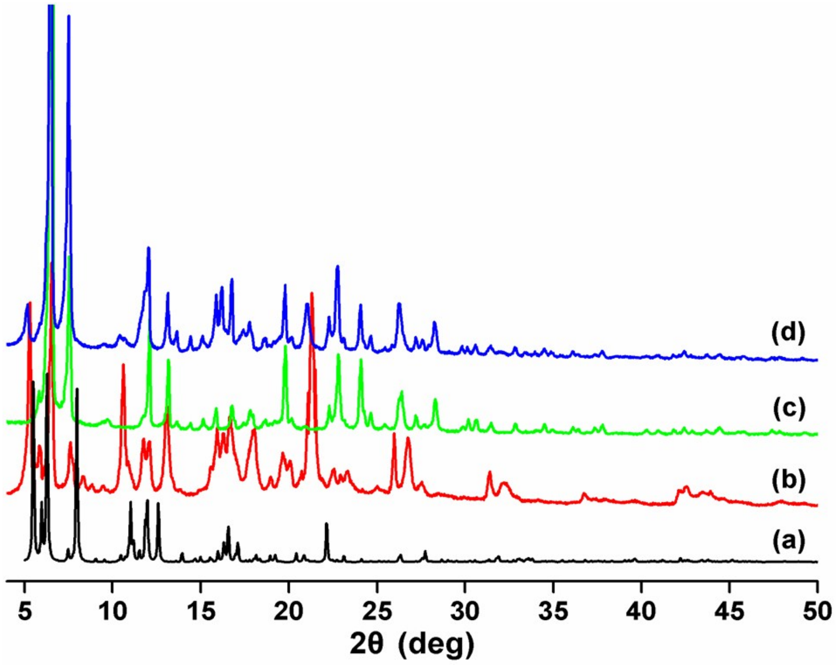

Figure S3. PXRD patterns of the (a) simulated single crystal data **1** at 153 K, (b) **1** at 298 K, (c) **1a** at 298 K, (d) DMF-regenerated sample **1'** at 298 K.

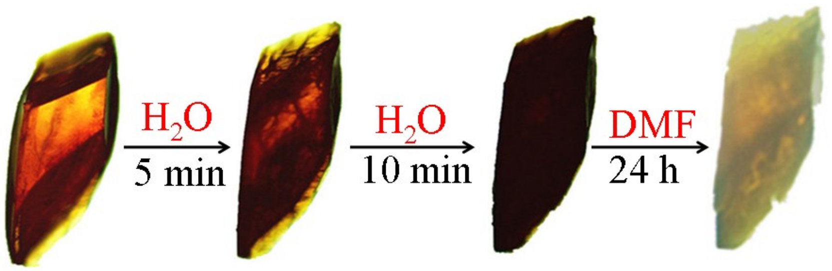

Figure S4. The images of **1** after soaking in H_2O for 5 min, 10 min and further soaking in DMF for 24 h.

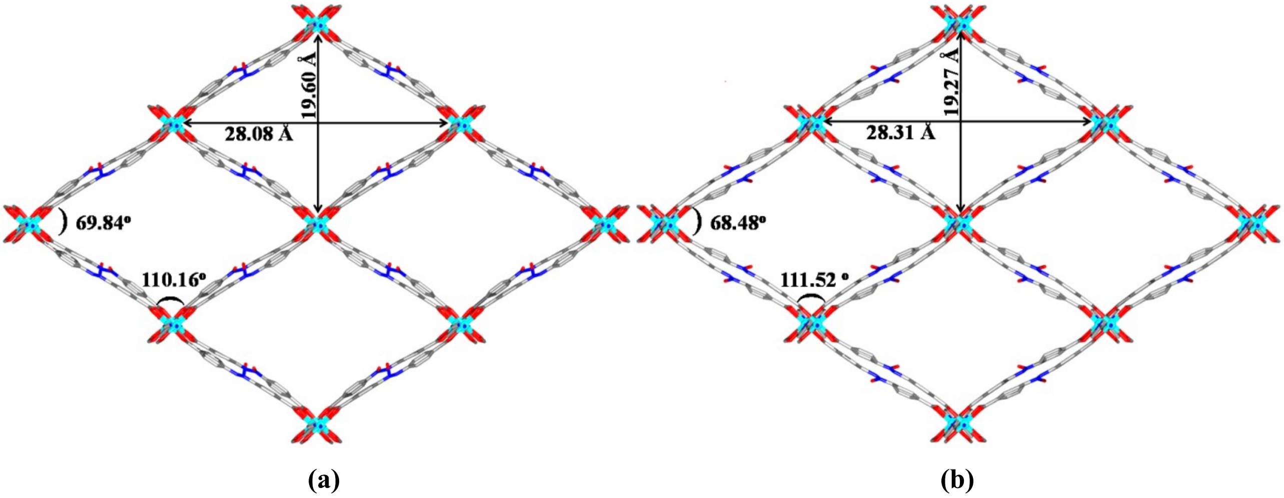

Figure S5. View of the 3D framework with 1D channels in (a) **1**, (b) **1**-$CHCl_3$ looking down the *c* axis.

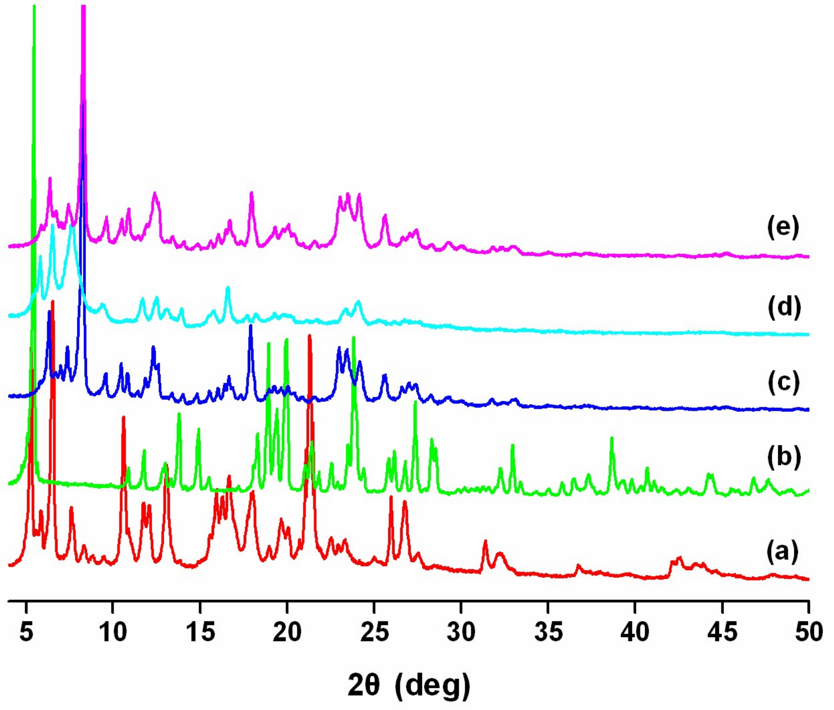

Figure S6. PXRD patterns of (a) **1**, (b) H_2O-exchanged **1** (soaking in water for 24 h, **1**-H_2O), (c) MeCN-exchanged **1** (soaking in MeCN for 24 h), (d) Acetone-exchanged **1** (soaking in acetone for 24 h), (e) $CHCl_3$-exchanged **1** (soaking in $CHCl_3$ for 24 h, **1**-$CHCl_3$).

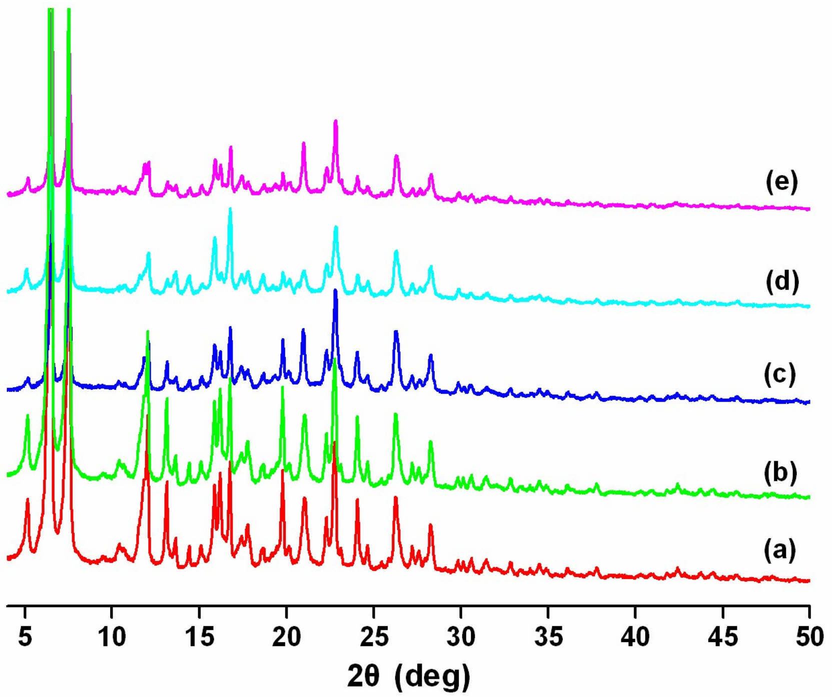

Figure S7. PXRD patterns of (a) DMF-regenerated sample **1'**, (b) H_2O-exchanged **1** after soaking in DMF for 24 h (**1**-H_2O-DMF), (c) MeCN-exchanged **1** after soaking in DMF for 24 h, (d) Acetone-exchanged **1** after soaking in DMF for 24 h, (e) $CHCl_3$-exchanged **1** after soaking in DMF for 24 h (**1**-$CHCl_3$-DMF).

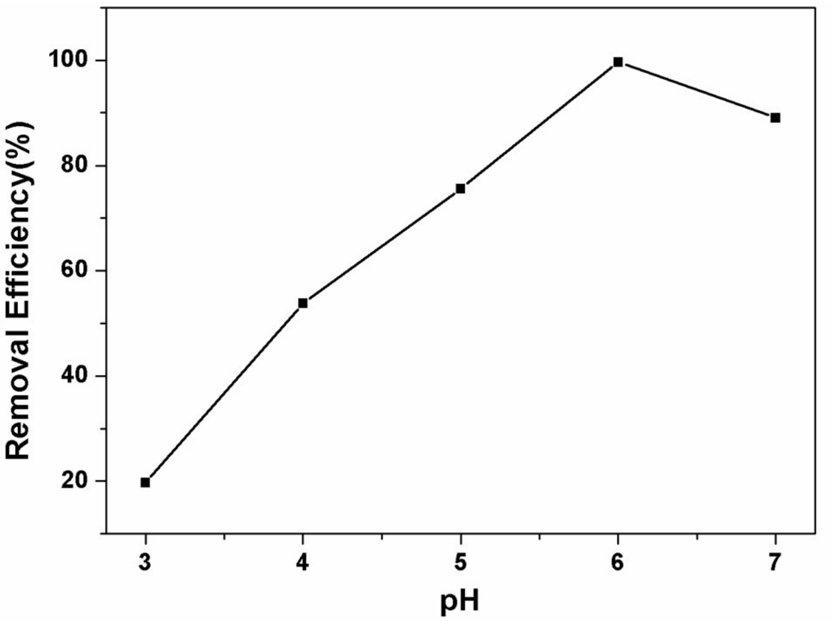

Figure S8. The effect of pH on the adsorption of Pb^{2+} for **1a**.

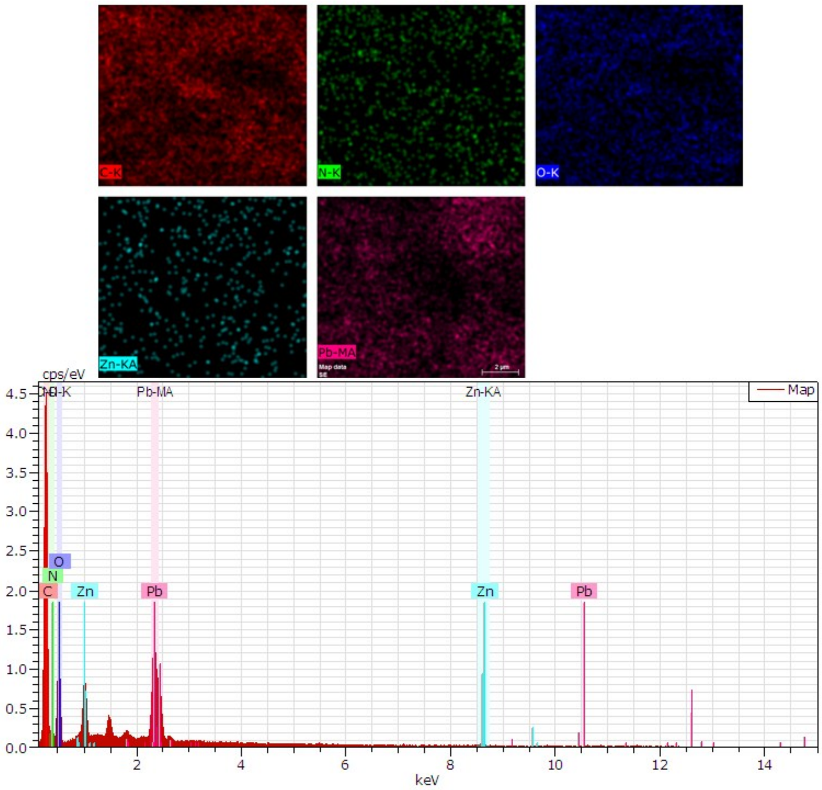

Figure S9. EDS spectra of **1a**-Pb.

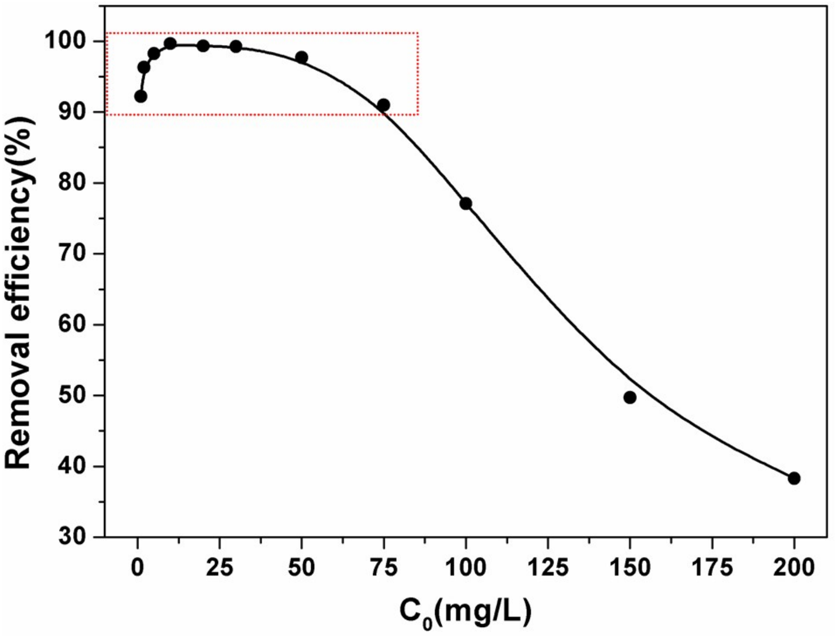

Figure S10. The removal ratio of **1a** toward Pb^{2+} at different initial concentration of Pb^{2+}.

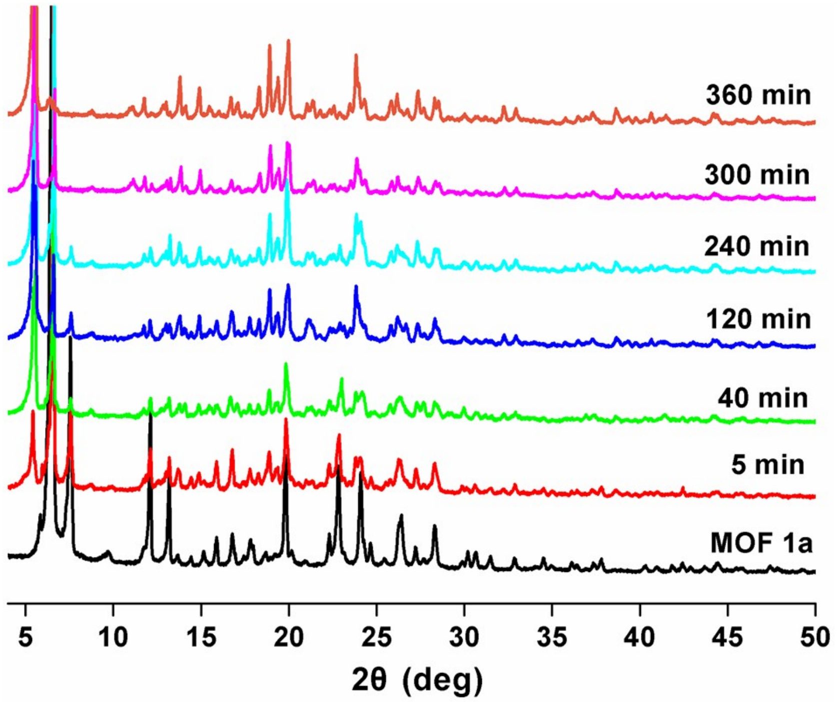

Figure S11. PXRD patterns of **1a** after soaking in Pb^{2+} solution for different time.

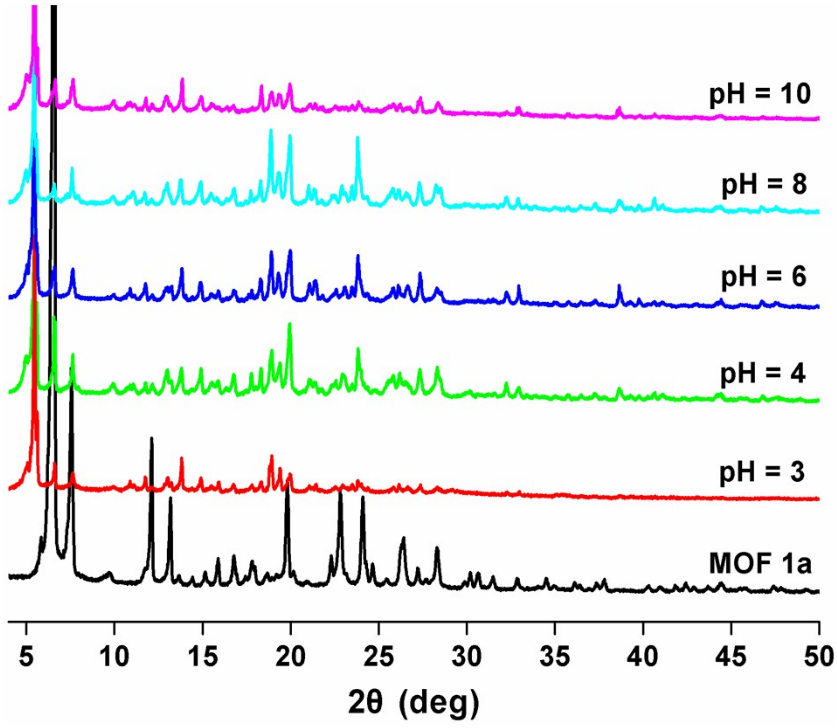

Figure S12. PXRD patterns of **1a** after immeresing in aqueous solution at pH = 3, 4, 6, 8, 10 (300 min).

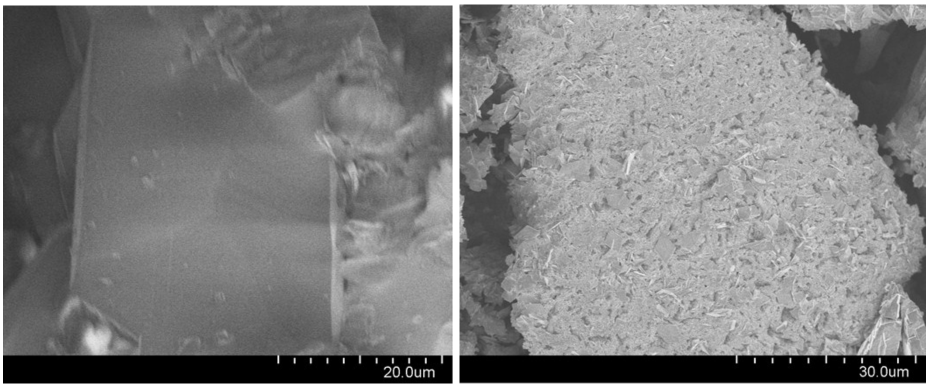

(**a**) (**b**)

Figure S13. (a) SEM image of **1a**. (b) SEM image of **1a**-Pb.

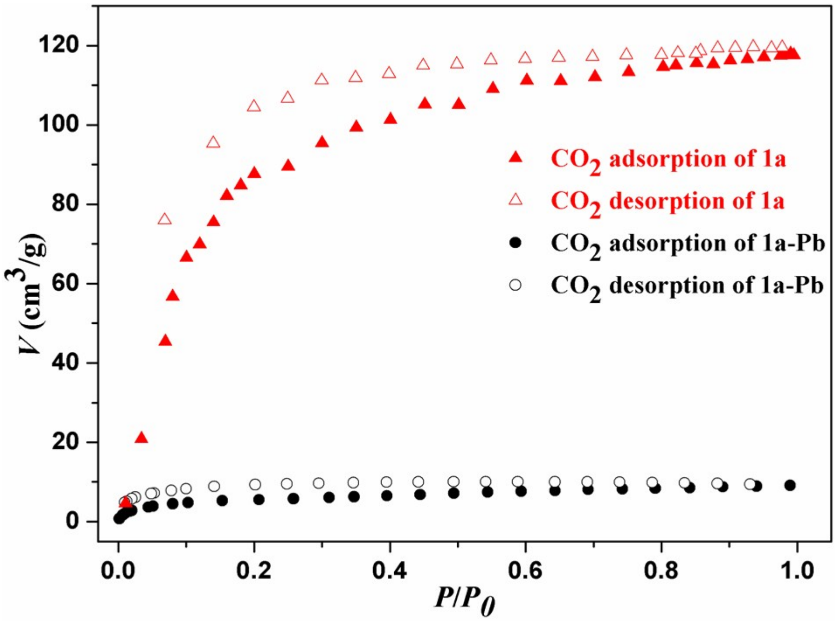

Figure S14. CO_2 adsorption and desorption isotherms of **1a** and **1a**-Pb.

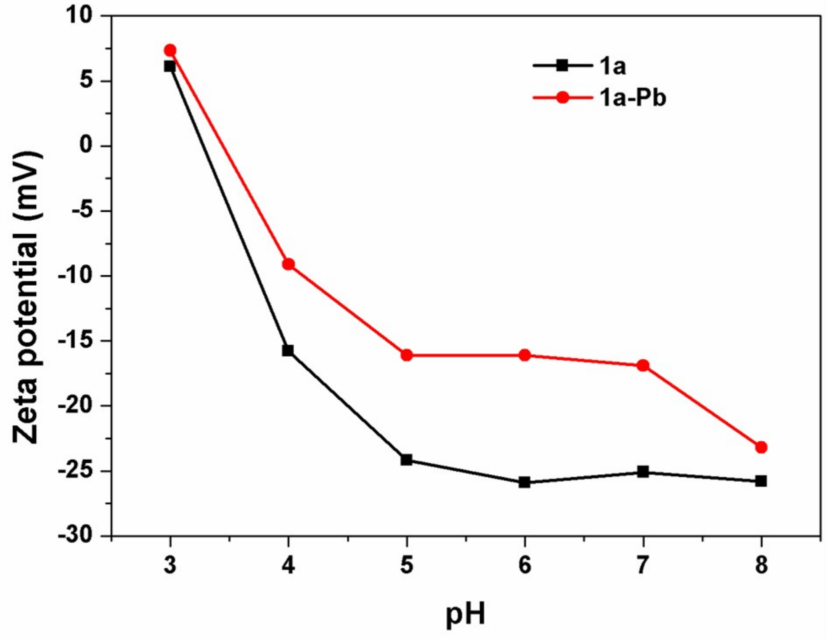

Figure S15. Zeta potentials of **1a** and **1a**-Pb.

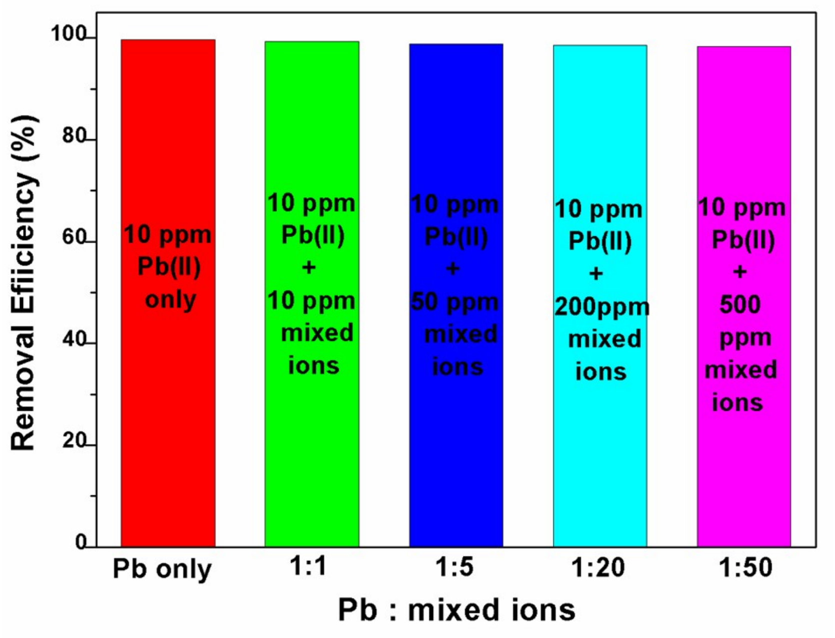

Figure S16. Influence of ionic strength on the Pb^{2+} adsorption by **1a**. (Initial Pb^{2+} concentration: 10 ppm; mixed ions: a mixed solution containing Na^{+}, Mg^{2+}, K^{+}, Ca^{2+}, Mn^{2+}, Co^{2+}, Ni^{2+}, Cd^{2+} with a certain initial concentration for each ion).

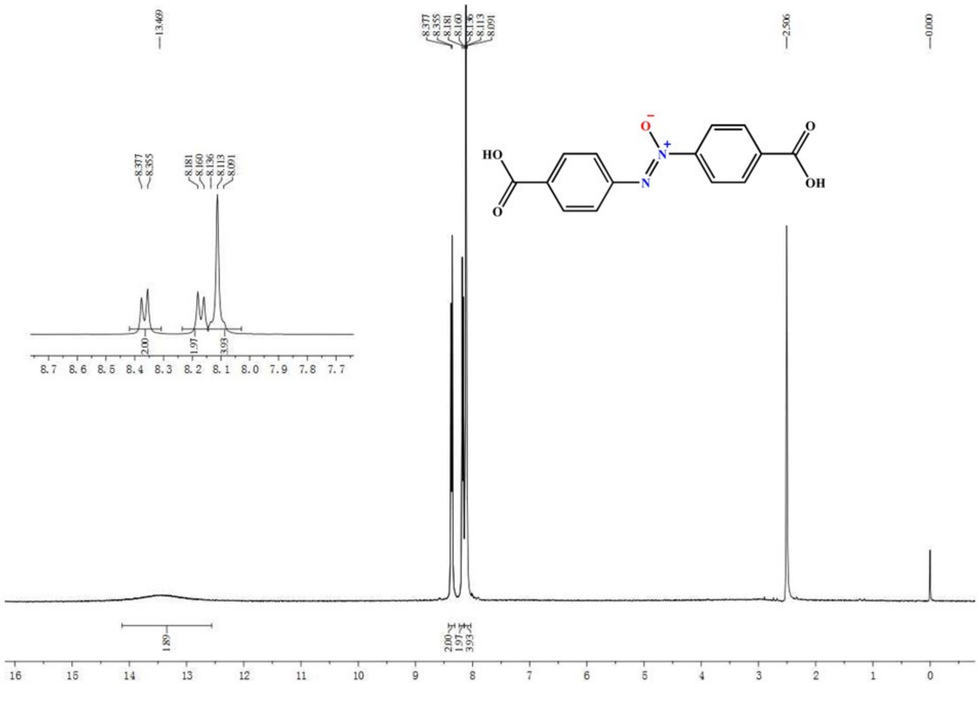

(**a**)

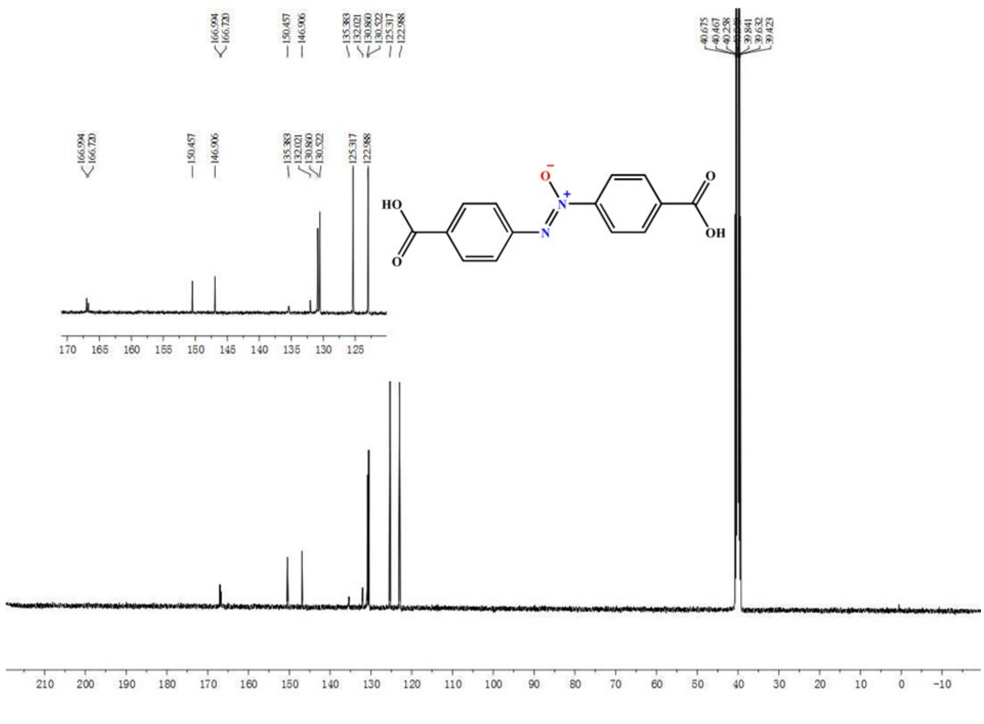

(**b**)

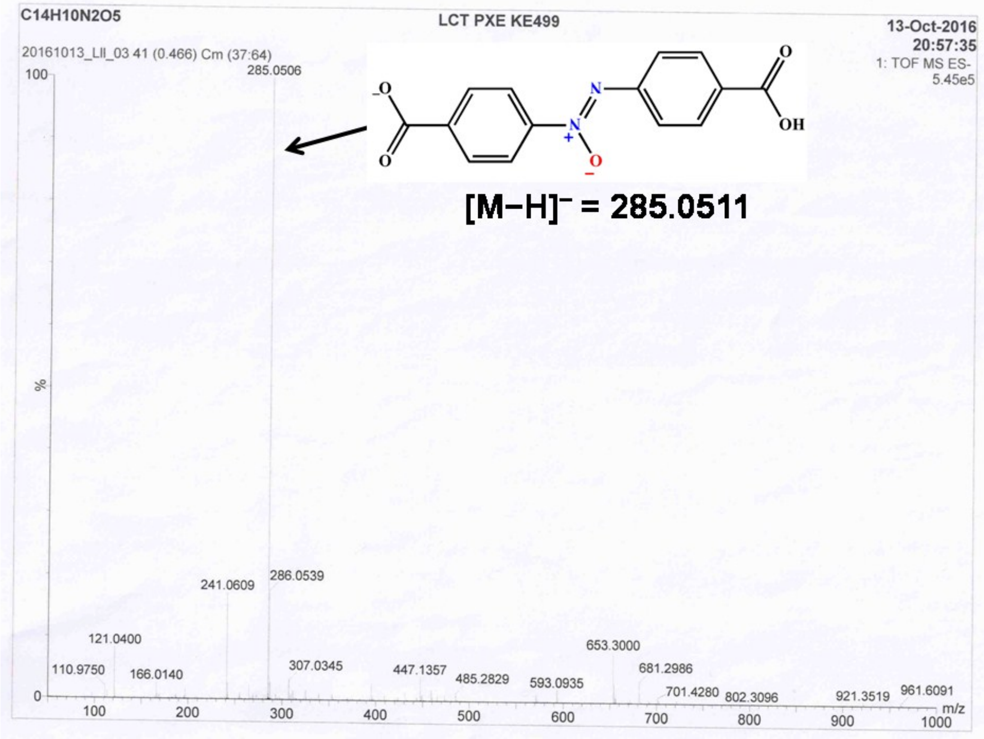

(**c**)

Figure S17. The (a) ^{1}H NMR, (b) ^{13}C NMR, (c) MS spectrum for **H_2L** ligand.

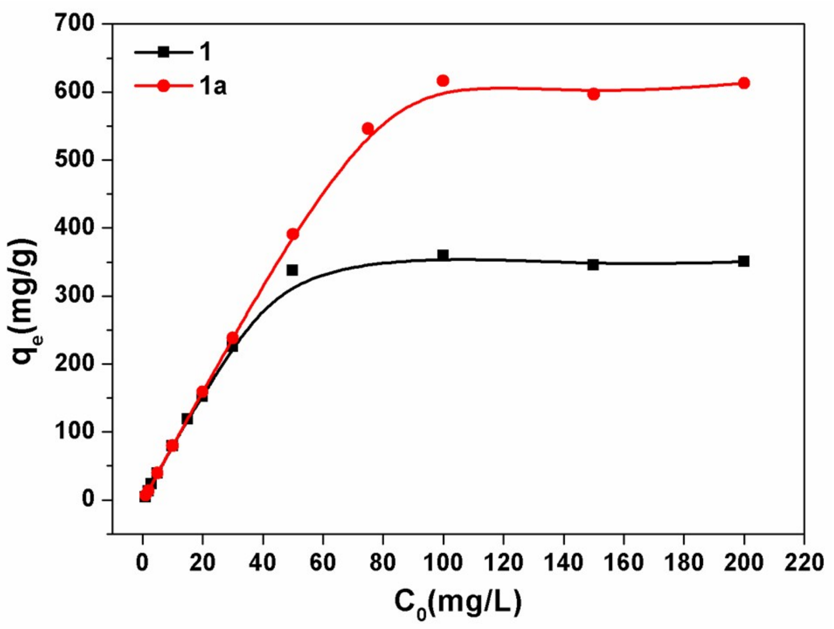

Figure S18. Pb^{2+} adsorption capacity at different concentrations for **1** and **1a**. There is almost no difference at low concentration range, but when the concentration above 50 ppm, the adsorption capacity of **1a** is obviously higher than that of **1**, and the difference increased along with the increasing concentration until achieving their saturated adsorption. The maximum adsorption capacity is 359.36 mg g^{-1}, which is obviously lower than that of **1a** (616.64 mg g^{-1}). Based on the adsorption behavior, we know that the activated sample **1a** give obviously better performance for Pb^{2+} sorption in the high concentration level. These results further confirm that the guest-free sample afford lager available space, which is responsible for the improved adsorption performance.

Table S1. Summary of unit cell parameters.

Parameters	**1**	**1**	**1**-$CHCl_3$	**1**-$CHCl_3$-DMF	**1**-H_2O-DMF	**1**-Pb-DMF
T (K)	153	298	153	298	298	298
a (Å)	19.604	21.79	19.268	21.73	21.18	22.41
b (Å)	28.079	26.94	28.308	26.96	27.18	26.67
c (Å)	32.670	33.00	32.705	32.95	32.83	33.11
α (°)	90	90	90	90	90	90
β (°)	94.47	92.24	95.62	92.64	93.25	92.38
γ (°)	90	90	90	90	90	90
V ($Å^3$)	17929	19358	17753	19288	18873	19772

Table S2. Summary of crystallographic data for **1** and **1**-$CHCl_3$.

Compound	**1**	**1**-$CHCl_3$
Empirical Formula	$C_{60}H_{39}N_9O_{15}Zn_3$	$C_{60}H_{39}N_9O_{15}Zn_3$
Formula Weight	1322.17	1322.17
Crystal System	monoclinic	monoclinic
Space Group	*C*2/*c*	*C*2/*c*
a (Å)	19.604(4)	19.268(4)
b (Å)	28.079(6)	28.308(6)
c (Å)	32.670(7)	32.705(7)
α (°)	90	90
β (°)	94.47(3)	95.62(3)
γ (°)	90	90
V (Å^3)	17929(7)	17753(7)
Z	8	8
T (K)	153	153
ρ_{calc} (g/cm^3)	0.980	0.989
F(000)	5376	5376
μ (MoKα, mm^{-1})	0.846	0.854
Total reflections	84732	49312
Unique reflections	15810 (R_{int} = 0.0546)	15624 (R_{int} = 0.1020)
No. of observations	10978	6385
No. of parameters	851	723
$R_1{}^a$	0.0623	0.1702
$wR_2{}^b$	0.1871	0.5157
GOF^c	1.093	1.523

^{a} $R_1 = \Sigma||F_o|-|F_c||/\Sigma|F_o|$. ^{b} $wR_2 = \{\Sigma w(F_o^2-F_c^2)^2/\Sigma w(F_o^2)^2\}^{1/2}$. ^{c} GOF = $\{\Sigma w(F_o^2-F_c^2)^2/(n-p)\}^{1/2}$, where n = number of reflections and p = total numbers of parameters refined.

Table S3. Comparison of maximum adsorption capacities (mg g^{-1}) with various adsorbents for removal of Pb^{2+}.

Adsorbents	Adsorption capacity (mg g^{-1})	References
MIL-101	15.78	1
ED-MIL-101	81.09	1
TMU-4	237	2
TMU-5	251	2
TMU-6	224	2
IOMN@NH_2-MIL-53(Al)	492.4	3
UiO-66-NH_2	232	4
Fe_3O_4/@HKUST-1/ DTz	104	5
HS-mSi@MOF-5	312.5	6
Fe_3O_4/@HKUST-1/ Pyridine	190	7
Ag-MOF	160	8
Nanocrystalline Zr-MOF	135.0	9
SH-Fe_3O_4/$Cu_3(BTC)_2$	198	10
MOF **1a**	616.64	this work

References

1. X. Luo, L. Ding and J. Luo, *J. Chem. Eng. Data*, 2015, **60**, 1732-1743.
2. E. Tahmasebi, M. Y. Masoomi, Y. Yamini and A. Morsali, *Inorg. Chem.*, 2015, **54**, 425-433.
3. R. Ricco, K. Konstas, M. J. Styles, J. J. Richardson, R. Babarao, K. Suzuki, P. Scopece and P. Falcaro, *J. Mater. Chem. A*, 2015, **3**, 19822-19831.
4. H. Saleem, U. Rafique and R. P. Davies, *Micropor. Mesopor. Mater.*, 2016, **221**, 238-244.
5. M. Taghizadeh, A. A. Asgharinezhad, M. Pooladi, M. Barzin, A. Abbaszadeh and A. Tadjarodi, *Microchim. Acta*, 2013, **180**, 1073-1084.
6. J. Zhang, Z. Xiong, C. Li and C. Wu, *J. Mol. Liq.*, 2016, **221**, 43-50.
7. M. R. Sohrabi, Z. Matbouie, A. A. Asgharinezhad and A. Dehghani, *Microchim. Acta*, 2013, **180**, 589-597.
8. M. Salarian, A. Ghanbarpour, M. Behbahani, S. Bagheri and A. Bagheri, *Microchim. Acta*, 2014, **181**, 999-1007.
9. N. Yin, K. Wang and Z. Li, *Chem. Lett.*, 2016, **45**, 625-627.
10. Y. Wang, H. Chen, J. Tang, G. Ye, H. Ge and X. Hu, *Food Chem.*, 2015, **181**, 191-197.
